# Outcomes of a combination of augmented MicroPulse and limited Continuous Wave Cyclophotocoagulation in patients with refractory glaucoma

**DOI:** 10.1007/s00417-021-05436-1

**Published:** 2021-10-25

**Authors:** Sanchay Gupta, Enchi Kristina Chang, Marika Chachanidze, Nathan Hall, Cameron Neeson, Emma Klug, Ta Chen Chang, David A. Solá-Del Valle

**Affiliations:** 1 Massachusetts Eye and Ear, Harvard Medical School, 243 Charles Street, Boston, MA 02114 USA; 2 Massachusetts Eye and Ear, Boston, MA USA; 3 Bascom Palmer Eye Institute, Miami, FL USA

**Keywords:** Glaucoma, Transscleral cyclophotocoagulation, Cyclophotocoagulation, Laser, Micropulse, Continuous wave

## Abstract

**Purpose:**

To assess the safety and effectiveness of augmented MicroPulse (MP-TSCPC) with limited Continuous Wave Transscleral Cyclophotocoagulation (CW-TSCPC) in patients with refractory glaucoma.

**Methods:**

Thirty-eight eyes of 38 patients underwent combined MP-TSCPC and CW-TSCPC at Massachusetts Eye and Ear. Kaplan–Meier survival curves and Wilcoxon paired sign rank tests were performed to evaluate intraocular pressure (IOP), glaucoma medication burden, best corrected visual acuity (BCVA), and adverse events.

**Results:**

With success defined as IOP reduction ≥ 30% and IOP between 5 and 18 mmHg, the cumulative probability of success at 1 year and 1.5 years were 0.81 (95% confidence interval (CI), 0.68–0.96) and 0.65 (95% CI, 0.50–0.86), respectively. With success defined as IOP reduction ≥ 50% and IOP between 5 and 18 mmHg, the success probability at 1 year and 1.5 years were 0.72 (95% CI, 0.57–0.89) and 0.56 (95% CI, 0.40–0.78), respectively. IOP and medication burden reductions were significant at all follow-up visits compared to baseline. Average IOP decreased from 27.9 mmHg at baseline to 11.4 mmHg at 1 year (*p* < 0.001) and 10.0 mmHg at 1.5 years (*p* < 0.001). Average medication burden decreased from 3.8 to 1.7 at 1.5 years (*p* = 0.001). No significant differences in visual acuity were observed at any time point. No long-term sight-threatening complications due to the combined procedure were observed, and most of the complications observed were mild and transient.

**Conclusion:**

In patients with refractory glaucoma, the combination of augmented MP-TSCPC with limited CW-TSCPC provides a significant IOP-lowering effect and decrease in medication burden without increased risk of postoperative complications.


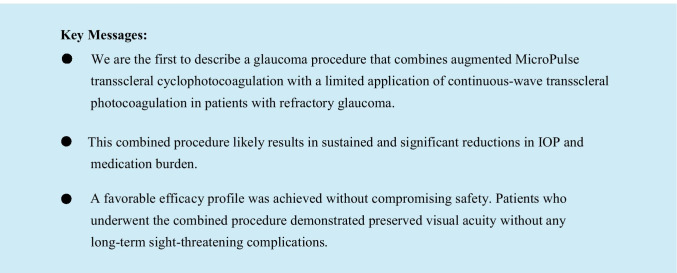


## Introduction

Transscleral cyclophotocoagulation (TSCPC) is used to lower intraocular pressure (IOP) in patients with advanced glaucoma, often as an alternative therapy to more invasive surgical procedures such as trabeculectomies and glaucoma drainage devices. Currently, two main methods of TSCPC are commonly used: Continuous Wave Transscleral Cyclophotocoagulation (CW-TSCPC) and MicroPulse Transscleral Cyclophotocoagulation (MP-TSCPC). In CW-TSCPC, destructive laser energy is delivered continuously to the ciliary body in an attempt to reduce aqueous humor production, while MP-TSCPC utilizes repetitive pulses of energy separated by periods of rest to achieve more targeted treatment of pigmented tissue in the ciliary processes and minimize collateral damage to neighboring tissues [1]. While both of these cyclophotocoagulation procedures represent a relatively targeted approach for reducing IOP, dissipated laser energy during administration may cause damage to surrounding tissues [1]. The extent of this dissipation may potentially underlie differences in postoperative complications that have been observed between these two procedures [1].

CW-TSCPC is known for its efficacy in reducing IOP and medication burden, which may depend on the magnitude of the preoperative IOP [2–7]. However, a significant risk of postoperative complications such as prolonged inflammation, vision loss, and phthisis bulbi has been reported along with a frequent need for retreatment [3, 8]. For instance, Egbert et al. noted vision loss in 23% (18/70) of patients who received CW-TSCPC [3]. Studies of MP-TSCPC have demonstrated fewer postoperative complications than CW-TSCPC and a need for multiple treatments over time [8, 9]. Given that MP-TSCPC was introduced more recently, the optimal MP-TSCPC settings to maximize outcomes have yet to be determined. Prior studies have suggested a possible dose-dependent relationship between power, number of treatments, and the IOP-reducing effect, which may be leveraged to optimize an IOP-reducing efficacy similar to CW-TSCPC [10, 11].

Although MP-TSCPC and CW-TSCPC have been investigated independently, there is currently no literature on the outcomes of these procedures performed in combination. If used in combination, the safety profile of MP-TSCPC could potentially be combined with the IOP-lowering efficacy of CW-TSCPC to provide better outcomes for glaucoma patients. Namely, a higher power used in MP-TSCPC combined with a limited application of CW-TSCPC may maximize IOP reduction without increasing the risk for adverse outcomes. In this retrospective case series, we describe our experiences with the safety and effectiveness of a combined approach using MP-TSCPC together with limited CW-TSCPC in patients with advanced glaucoma.

## Methods

### Study design

Following approval by the Massachusetts General Brigham Institutional Review Board, a retrospective review of medical records was conducted to identify patients who underwent simultaneous MP-TSCPC and CW-TSCPC at the Massachusetts Eye and Ear. These procedures were performed by a single provider between April 2018 and November 2019 after patients were appropriately consented for the intervention. Data collection methods abided by the Declaration of Helsinki and the Health Portability and Accountability Act. As this was a retrospective study of medical records, informed consent was not required. Medical records of patients under a single provider were identified using financial claims data (Current Procedural Terminology codes 66,710, 66,711), and all identified records were reviewed. Inclusion criteria for glaucoma patients in this chart review were as follows: (1) concurrent MP-TSCPC and CW-TSCPC were performed on the same eye in the same day; (2) preoperative IOP > 20 mmHg OR ≥ 3 glaucoma medications were needed preoperatively; and (3) failed prior IOP-lowering glaucoma procedure OR documented medication side effect OR suboptimal visual acuity unlikely to improve. Patients were excluded if the affected eye had mild glaucoma or if best-corrected visual acuity (BCVA) was better than 20/30. No exclusions were made based on the type of glaucoma.

Preoperative data collected included patient age, gender, glaucoma diagnosis and stage, previous ocular surgeries, IOP, number of glaucoma medications, and visual acuity (VA). Glaucoma stages were defined using optical coherence tomography (OCT) and Humphrey visual field (HVF) findings as previously described by Fellman et al. [12]. A diagnosis of moderate glaucoma was conferred in eyes with (1) optic nerve abnormalities consistent with glaucoma (e.g., circumpapillary retinal nerve fiber layer thinning on OCT) and (2) glaucomatous visual field abnormalities in a single hemifield and not within 5 degrees of fixation [12]. Severe glaucoma was defined by (1) optic nerve abnormalities consistent with glaucoma and (2) glaucomatous visual field abnormalities in both hemifields and within 5 degrees of fixation in at least one hemifield [12]. Patients were staged as indeterminate if their visual field data was unreliable or unavailable (e.g., no light perception, count fingers vision) [12].

IOP was measured by the same glaucoma specialist every time using Goldmann applanation tonometry. Preoperative IOP and number of medications were calculated as an average of the values from the two consecutive visits prior to the procedure. Intraoperative data collected included laser settings such as power, duration, and sites treated, as well as any intraoperative complications. Data was collected from follow-up visits at 1 day (POD1), 2 weeks (POW2), 6 weeks (POW6), 3 months (POM3), 6 months (POM6), 1 year (POY1), and 1.5 years (POY1.5) using standard follow-up time windows outlined in the World Glaucoma Association consensus document for reporting glaucoma surgical trials [13]. Postoperative data included IOP, number of glaucoma medications, VA, duration of follow-up, subsequent IOP-lowering procedures, and the presence of postoperative complications such as inflammation or fibrin in the anterior chamber (AC), hypotony, or cystoid macular edema (CME). Data collection was stopped on the date of the second glaucoma procedure for patients who received a second glaucoma procedure, thus limiting the follow-up length of these patients to the date of the second procedure.

### Surgical procedure

MP-TSCPC was performed with IRIDEX’s Generation 1 MicroPulse P3 glaucoma device (MP3) and CW-TSCPC with IRIDEX’s G-Probe device (IRIDEX Corp., Mountain View, CA). The IRIDEX G6 810-nm laser (IRIDEX, CYCLO G6; Glaucoma Laser System, Mountain View, CA) was used with both devices. All procedures were performed by the same glaucoma specialist. Informed consent was obtained from all patients for the combined MP-TSCPC and CW-TSCPC procedure.

A retrobulbar block was administered by anesthesia with 5 mL of 1% preservative-free lidocaine and 0.375% preservative-free bupivacaine, along with monitored anesthesia care. The MP3 probe was applied perpendicularly 2 mm from the limbus on the adjacent sclera. Treatment settings for MP-TSCPC were 2000–2400 mW at 31.3% duty power applied to the superior and inferior hemispheres for 180 s each. Ninety seconds of a sweeping technique were followed by 90 s of a stop-and-continue technique. The sweeping technique consisted of a slow, continuous sliding motion in an arc along the limbus, avoiding the 3 and 9 o’clock positions. The exact number of sweeps was not recorded. The stop-and-continue technique was performed by dividing each hemisphere along the limbus into 9 equal sections and applying a 10 s burn to each section. Augmented MP-TSCPC was performed as previously described by Nirappel et al. [11].

Following augmented MP-TSCPC, a limited procedure with CW-TSCPC was performed with the G-probe. The G-probe was placed perpendicular to the curve of the limbus on the adjacent sclera. Treatment settings were 950–2300 mW of power for 3–4 s per burn for approximately 3–5 burns per hemisphere depending on the amount of IOP reduction needed and the area of viable conjunctiva. The power was started at 950 mW and up-titrated in 200 mW increments to a maximum of 2300 mW or until one or two “pops” were audible. If “pops” were heard, the power was titrated down. The 3 and 9 o’clock positions were again avoided to minimize risk of damage to the long posterior vessels and nerves. Copious balanced saline solution was used as the coupling agent for both procedures.

Immediately after the procedure, all patients received a subconjunctival injection of 1–2 mL of 0.5 mg dexamethasone with antibiotics. Maxitrol ointment was placed on the eye before it was patched and shielded. Uveitic patients received 1 g intravenous methylprednisolone immediately postoperatively. Otherwise, on POD1, patients were started on topical prednisolone acetate 1% and/or ketorolac 0.5%. Difluprednate 0.05% or fluorometholone 0.1%/loteprednol 0.5% was substituted as necessary depending on the amount of inflammation present. Drops were typically used 4 times daily for the first week and tapered by 1 drop daily for each subsequent week. Individual tapering plans were modified at the surgeon’s discretion based on the patient’s degree of inflammation at the follow-up visits.

### Outcome measures

Primary outcome measures were the average reduction in IOP, number of glaucoma medications, and Kaplan–Meier (KM) success probabilities at each postoperative follow-up visit. For survival analysis, success was defined as an IOP between 5 and 18 mmHg with either a ≥ 30% or ≥ 50% IOP reduction from baseline regardless of glaucoma medication use. Treatment failure was defined as an inability to meet the specified success criteria on two consecutive follow-up visits, with the latter of the two dates as the failure date. This was done to avoid confounding from temporary intraocular pressure fluctuations. Patients were included in our survival analyses if they had a minimum of 3 months of follow-up without failing to achieve our success criteria in order to avoid failures resulting from short-term postoperative IOP fluctuations. Patients who required an additional glaucoma procedure after the initial procedure or developed no light perception vision were considered failures for survival analysis, and their data was censored for additional analyses. Secondary outcome measures included visual acuity and complication rates.

### Statistical analysis

Statistical analysis was performed using R (version 3.6.2) and GraphPad Prism (version 8.3.1, San Diego, CA). Kaplan–Meier survival curves were generated to represent survival rates based on predefined criteria for success. A multivariate, fully adjusted Cox PH regression model was fit to adjust for and obtain hazard ratios for baseline characteristics. For preoperative and postoperative comparisons, the Wilcoxon paired signed rank test was used for data following a non-normal distribution (IOP, number of medications, VA) and paired t-tests for data following a normal distribution at postoperative day 1 (POD1), postoperative week 2 (POW2), postoperative week 6 (POW6), postoperative month 3 (POM3), postoperative month 6 (POM6), postoperative year 1 (POY1), and postoperative year 1.5 (POY1.5). For VA analysis, Snellen visual acuities measured in clinic were converted to logarithm of minimum angle of resolution (LogMAR) equivalents. Count fingers (CF) vision was represented by 2 on the logMAR scale (20/2000), and hand motion (HM) vision was represented by 3 (20/20000). Patients with light perception and no light perception vision were not converted to logMAR equivalents and excluded from mean calculations and paired t-testing. Statistical significance was defined as *p* < 0.05. Overall, statistical analysis was conducted as previously described by Chang et al. [14].

## Results

### Demographics and preoperative data

Data were obtained from 38 eyes of 38 patients who received both MP-TSCPC and CW-TSCPC in a single session. Sample sizes for follow-ups were smaller than the original cohort due to loss-to-follow-up or rescheduling of visits to future dates that did not fit our timeline. The average follow-up period was 16.3 ± 9.6 months with a range of 3–34.3 months. Demographic and baseline characteristics are summarized in Table 1.

**Table 1:** Demographic and ocular data

Parameters		Parameters	
*Demographics*		*Preoperative baseline*	
Eyes, *N*	38	IOP (mm Hg)	
Female sex, *N* (%)	15 (39.5)	Mean ± SD	27.9 ± 8.2
Age (Year)		Range	11–47
Mean ± SD	69.2 ± 17.3	Visual acuity	
Range	30–97	Range	LP – 20/30
		# of glaucoma medications	
*Glaucoma stage, N (%)*		Mean ± SD	3.8 ± 1.2
Moderate	5 (13.2)	Mean HVF deviation	
Severe	31 (81.6)	Mean ± SD*	− 20.5 ± 8.5
Indeterminate	2 (5.3)	Range*	− 30.9–0.12
		Prior glaucoma laser, *N* (%)	
*Glaucoma type, N (%)*		None	12 (31.6)
Mixed mechanism^a^	19 (50.0)	SLT	7 (18.4)
Primary open angle	9 (23.7)	LPI	4 (10.5)
Neovascular	4 (10.5)	MPCPC/CPC	14 (36.8)
Pseudoexfoliation	2 (5.3)	Prior glaucoma surgery, *N* (%)	
Juvenile open angle	2 (5.3)	None	1 (2.6)
Uveitic	1 (2.6)	Trabeculectomy	4 (10.5)
Chronic angle closure	1 (2.6)	Tube shunt	14 (36.8)
		PEcK, iStent^b^	3 (7.9)
		Other (PPV, DSEK, Phaco)	16 (42.1)
		Lens status, *N* (%)	
		Phakic	5 (13.2)
		Pseudophakic	30 (78.9)
		Aphakic	3 (7.9)

*N* number of eyes, *IOP* intraocular pressure, *SLT* selective laser trabeculoplasty, *LPI* laser peripheral iridotomy, *MPCPC* micropulse cyclophotocoagulation, *CPC* cyclophotocoagulation, *PEcK* phacoemulsification and endoscopic cyclophotocoagulation with Kahook Dual Blade, *PPV* pars plana vitrectomy, *DSEK* Descemet stripping endothelial keratoplasty, *HVF* Humphrey Visual Field

^a^Mixed mechanism glaucoma includes a combination of primary open angle, chronic angle closure, steroid response, pseudoexfoliative, traumatic, uveitic, and neovascular glaucoma as well as glaucoma secondary to an iris melanoma or corneal transplantation

^b^iStent Trabecular Micro-Bypass Stent (Models GTS100R and GTS100L, Glaukos Corporation, San Clemente, California) and iStent *inject®* Trabecular Micro-Bypass System (Model G2-M-IS, Glaukos Corporation, San Clemente, California)

^*^Unreliable visual fields and patients unable to complete visual field testing due to poor vision (light perception, hand motion) are not represented

### Surgical data

A summary of TSCPC laser settings is listed in Table 2. For MP-TSCPC, the average power was 2189.5 ± 100.8 mW for 180 s per hemisphere. For CW-TSCPC, an average of 8.1 ± 3.6 spots were applied to the limbus at power settings of 1807.9 ± 311.8 mW for 3–4 s per spot. Two patients were treated in a single hemisphere with only 3 spots for CW-TSCPC. For these two patients, one patient had a melanoma in the untreated hemisphere, and the other had thin conjunctiva secondary to prior trabeculectomy and Ahmed glaucoma implant surgery.

**Table 2:** TSCPC laser settings

Parameters	
MP-TSCPC	
Power (mW, mean (SD))	2189.5 (100.8)
Duration of treatment per hemisphere (s, mean (SD))	180 (0)
CW-TSCPC	
Power (mW, mean (SD))	1807.9 (311.8)
Median number of treatment spots	7.5
Mean number of treatment spots (SD)	8.1 (3.6)
Range of number of treatment spots	3–12
Range of spot duration per hemisphere (s)	3–4

*MP-TSCPC* MicroPulse Transscleral Cyclophotocoagulation, *CW-TSCPC* continuous wave transscleral cyclophotocoagulation, *SD* standard deviation, *mW* milliwatts, *s* seconds

Three patients underwent additional glaucoma surgery, and two patients received additional cyclophotocoagulation after the combined procedure. These patients were all censored from analyses after those events. Two patients achieved successful IOP reduction after the combined procedure but underwent glaucoma valve revision at POM5 and POM13 due to tube migration.

### Effectiveness

**Fig. 1:**
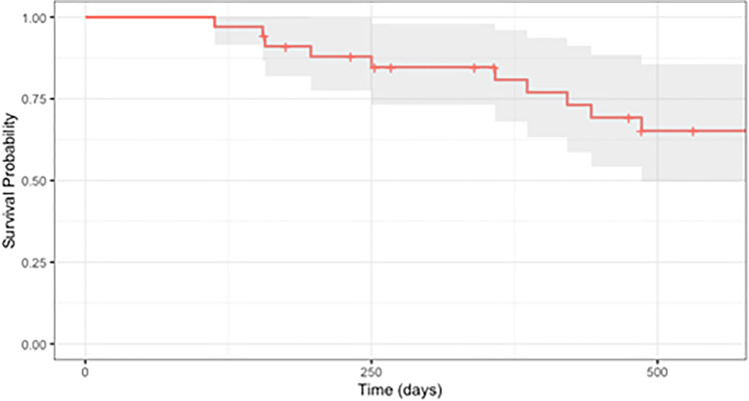
Kaplan–Meier survival curve of combined MicroPulse Transscleral Photocoagulation (MP-TSCPC) and Continuous Wave Transscleral Cyclophotocoagulation (CW-TSCPC), where failure was defined by an intraocular pressure (IOP) < 6 mmHg or > 18 mmHg or a < 30% decrease from baseline on at least two consecutive follow-up visits after 3 months or need for additional surgery to control IOP

**Fig. 2:**
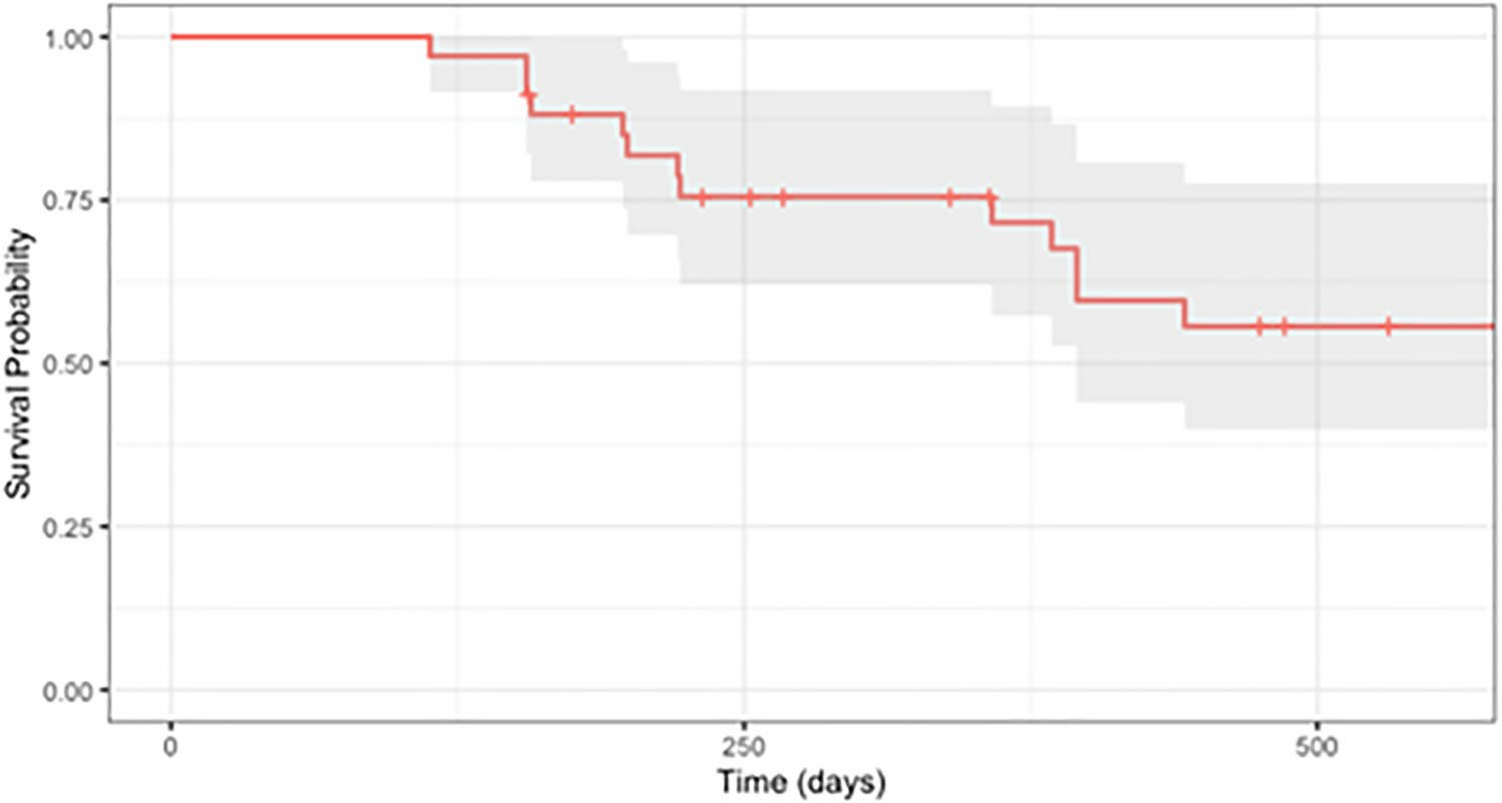
Kaplan–Meier survival curve of combined MicroPulse Transscleral Photocoagulation (MP-TSCPC) and Continuous Wave Transscleral Cyclophotocoagulation (CW-TSCPC), where failure was defined by an intraocular pressure (IOP) < 6 mmHg or > 18 mmHg or a < 50% decrease from baseline on at least two consecutive follow-up visits after 3 months or need for additional surgery to control IOP

Kaplan–Meier survival curves based on our criteria for success are depicted in Fig. 1 and Fig. 2. Survival probabilities at follow-up time points of interest are listed in Table 3. With success defined as IOP between 5 and 18 mmHg and ≥ 30% IOP reduction, cumulative probability of success was 0.81 (95% CI, 0.68–0.96) at 1 year and 0.65 (95% CI, 0.50–0.86) at 1.5 years postoperatively; when success was defined as an IOP reduction ≥ 50% with IOP between 5 and 18 mmHg, cumulative probability of success was 0.72 (95% CI, 0.57–0.89) at 1 year and 0.56 (95% CI, 0.40–0.78) at 1.5 years postoperatively.

**Table 3:** Life table

	IOP reduction ≥ 30% and 5 < IOP ≤ 18 mm Hg	IOP reduction ≥ 50% and 5 < IOP ≤ 18 mm Hg
	Cumulative success (%) ± SE (95% confidence interval)	Cumulative success (%) ± SE (95% confidence interval)
3 months	100.0 ± 100.0 (100.0, 100.0)	100.0 ± 100.0 (100.0, 100.0)
* N*	34	34
6 months	91.1 ± 4.9 (81.9, 100.0)	88.1 ± 5.6 (77.9, 99.8)
* N*	32	32
1 year	80.8 ± 7.1 (68.0, 96.0)	71.6 ± 8.1 (57.3, 89.4)
* N*	24	23
1.5 years	65.2 ± 9.1 (49.7, 85.6)	55.7 ± 9.4 (39.9, 77.6)
* N*	18	15

*IOP* intraocular pressure, *SE* standard error, *N* number of eyes

With success defined as ≥ 50% IOP reduction, the hazard ratio derived from multivariate Cox proportional hazard regression analyses for preoperative IOP was statistically significant at 0.85 (95% CI 0.77–0.95, *p* = 0.003), representing a 15% decrease in the hazard of failure. With success defined as ≥ 30% IOP reduction, the hazard ratio derived from Cox proportional hazard regression analyses for preoperative IOP was also statistically significant at 0.86 (95% CI 0.76–0.97, *p* = 0.01), representing a 14% decrease in the hazard of failure. Hazard ratios for age, race, sex, family history, glaucoma stage, preoperative medication burden, and postoperative steroid or NSAID use for both success criteria were not statistically significant.

Postoperative IOP was significantly lower than preoperative IOP at all follow-up time points (Table 4). The average baseline IOP was 27.9 ± 8.2 mmHg with an average of 3.8 ± 1.2 medications. At postoperative month 3, IOP was reduced from baseline by an average of 14.8 ± 10.0 mmHg. Moreover, this IOP reduction persisted over time, with study participants demonstrating an average IOP reduction of 15.1 ± 8.9 mmHg and 17.2 ± 8.6 mmHg at POY1 and POY1.5 from baseline, respectively (*p* < 0.001).

**Table 4:** Postoperative outcomes data

	IOP (mm Hg)	Medications	Visual acuity (LogMAR)^a^
Preoperative (*n* = *38*)			
Mean (SD)	27.9 (8.2)	3.8 (1.2)	1.65 (1.08)
Median	25.5	4.0	1.30
1 day (*n* = *37*)			
Mean (SD)	15.7 (5.3)	0.5 (1.3)	1.65 (1.01)
Decrease from baseline	12.0 (8.1)	3.4 (1.6)	− 0.10 (0.54)
Median	16.0	0	1.30
* p* value	< 0.001*	< 0.001*	0.360
2 weeks (*n* = *35*)			
Mean (SD)	9.0 (4.4)	2.3 (1.6)	1.58 (0.91)
Decrease from baseline	18.3 (8.7)	1.6 (1.5)	0.01 (0.67)
Median	8.0	2.0	1.30
* p* value	< 0.001*	< 0.001*	0.976
6 weeks (*n* = *30*)			
Mean (SD)	10.3 (4.3)	2.2 (1.7)	1.56 (0.96)
Decrease from baseline	17.6 (10.1)	1.9 (1.6)	0.12 (0.49)
Median	10.5	2.3	1.29
* p* value	< 0.001*	< 0.001*	0.184
3 months (*n* = *26*)			
Mean (SD)	12.6 (7.2)	1.9 (1.5)	1.69 (0.98)
Decrease from baseline	14.8 (10.0)	2.1 (1.6)	− 0.12 (0.46)
Median	10.5	2.0	1.48
* p* value	< 0.001*	< 0.001*	0.209
6 months (*n* = *30*)			
Mean (SD)	11.3 (4.1)	1.9 (1.5)	1.44 (0.97)
Decrease from baseline	16.5 (9.9)	2.0 (1.6)	0.10 (0.45)
Median	11.8	2.0	1.25
* p* value	< 0.001*	< 0.001*	0.962
1 year (*n* = *23*)			
Mean (SD)	11.4 (5.0)	2.1 (1.6)	1.48 (1.97)
Decrease from baseline	15.1 (8.9)	1.9 (1.8)	− 0.10 (0.54)
Median	11.0	2.0	1.18
* p* value	< 0.001*	< 0.001*	0.313
1.5 years (*n* = *19*)			
Mean (SD)	10.0 (3.5)	1.7 (1.4)	1.45 (1.01)
Decrease from baseline	17.2 (8.6)	2.3 (2.0)	0.11 (0.58)
Median	10.0	2.0	1.18
* p* value	< 0.001*	0.001*	0.721

^a^Patients with LP or NLP at these time points were excluded in mean and *p* value calculations due to a lack of a validated LogMAR equivalent

*Indicates significant *p* value < 0.05

**Fig. 3:**
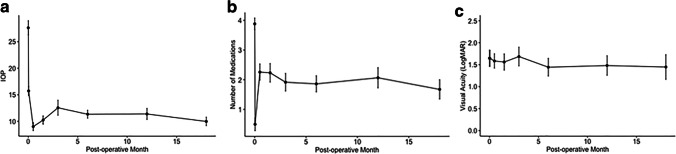
Line graph of average values of postoperative **a** intraocular pressure, **b** number of medications, and **c** visual acuity over time, with error bars denoting standard error of the mean

Postoperative glaucoma medication burden was also significantly lower than preoperative medication burden at all follow-up time points (Table 4). At POY1.5, the mean number of IOP lowering medications was 1.7 ± 1.4 (compared to 3.8 ± 1.2 preoperatively; p = 0.001). Figure 3 illustrates the mean number of IOP lowering medications used by the study cohort at each follow-up visit.

With respect to LogMAR visual acuity, there were no statistically significant differences from baseline VA at any of the postoperative time points (Table 4), demonstrating preservation of visual acuity across study participants for the duration of follow-up.

### Safety

Postoperative complication rates are listed in Table 5. Hyphema and evidence of fibrin noted shortly after the procedure resolved in all patients by POW2. Seven patients (20%) developed hypotony by POW2 that subsequently resolved by POW6 in 6 patients and at POM6 in 1 patient. Two patients experienced transient hypotony at POM6 that resolved by the next follow-up and one patient developed hypotony at POM16 (this patient developed NLP vision due to a choroidal melanoma). Importantly, there was no evidence of choroidal effusion, hypotony maculopathy, or phthisis bulbi in any of these patients throughout the postoperative period. One patient with a malignant iris melanoma developed a small hyphema at POW9 that persisted until POM9. By POM3, anterior chamber inflammation was present in only one patient (4.0%), which subsequently resolved by POY1. Two patients developed new anterior chamber inflammation several months after the combined procedure at POM9 and POY1 in the setting of repeat procedures. One patient was followed by our retina service for CME prior to the procedure, and the CME persisted throughout POY1.5. Another patient developed CME at 3 months, which persisted and was observed at POY1 but did not affect baseline visual acuity. The same patient required tube revision at 13 months due to tube-corneal touch.

**Table 5:** Complication rates

	*N* (%)				
	AC inflammation	Hyphema	Fibrin	Hypotony	Cystoid macular edema
Total (*n* = 38)	23 (60.5)	3 (7.9)	4 (10.5)	10 (26.3)	2 (5.3)
Early^a^ (*n* = 38)	22 (57.9)	3 (7.9)	4 (10.5)	7 (18.4)	2 (5.3)
Late^b^ (*n* = 32)	3 (9.4)	1 (3.1)	0 (0.0)	4 (12.5)	2 (6.3)

*N* number of eyes in group, *n* total number of eyes, *AC* anterior chamber

^a^Complications present up to 3 months postoperatively, not including preoperative findings

^b^Complications present after 3 months postoperatively

## Discussion

In this study, we examined the clinical outcomes of a procedure that augments MP-TSCPC with limited CW-TSCPC in patients with refractory glaucoma. Results from this study suggest that the combined procedure is effective in lowering IOP and medication burden without compromising safety. Our data demonstrated a significant IOP reduction from baseline sustained through POY1.5 in a majority of cases, along with a significant reduction in medication burden over this period. Visual acuity was unchanged from baseline at all postoperative visits, suggestive of no long-term visual deficits from this procedure.

Despite the elevated risk of postoperative complications widely reported in CW-TSCPC compared to MP-TSCPC, complication rates in our study were lower than or comparable to previously reported complication rates following CW-TSCPC. In a review of 7 studies of CW-TSCPC, Souissi et al. reports chronic uveitis in 10.9% of patients [15]. In our study, AC inflammation following the combined procedure resolved in almost all cases during the early postoperative period, and transient AC inflammation in 3 other patients was either attributable to the additional glaucoma procedures they received or resolved spontaneously. Our rate of hypotony was lower than that reported in many studies of CW-TSPC, with rates ranging from 1.1 to as high as 39% in treated eyes [8, 16–19].

Singh et al., Chen et al., and Yildirim et al. report rates of hyphema in 1.1%, 12.0%, and 15.0% of patients following CW-TSCPC [20–22]. In our study, 7.9% of patients developed hyphema in the early postoperative follow-up period that spontaneously resolved in all but one patient with a concurrent diagnosis of malignant iris melanoma. It is difficult to conclude whether the onset of hyphema in this patient was related to the procedure itself or worsening ocular malignancy, though its incidence at POM3 is potentially suggestive of the latter. Additionally, CME rates of 2–12% for CW-TSCPC have been reported previously in the literature [7, 15, 23]. Given that 1 patient had CME prior to our operation, our incidence of CME in a single patient is on the lower end of these rates.

Considering that traditional CW-TSCPC is performed with a much higher number of burns than in our procedure (i.e., 20–28 burns vs. 5–12 burns), the lower risk of adverse outcomes in our study may be attributed to the lower total continuous laser energy delivered and burn area [8]. Furthermore, a review of MP-TSCPC studies reveals that augmenting MP-TSCPC with limited application of CW-TSCPC leads to a similar safety profile as performing MP-TSCPC alone. Emanuel et al., Nirappel et al., and Kuchar et al. report rates of hypotony following MP-TSCPC in 7.1%, 11.8%, and 26.3% of eyes [11, 24, 25]. Studies of MP-TSCPC have noted anterior chamber inflammation in up to 48.8% of eyes [24]. As mentioned earlier, we did note the incidence of CME in one patient which was not reported in the above studies of MP-TSCPC. However, this did not compromise vision. Thus, our results suggest that augmenting MP-TSCPC with limited CW-TSCPC is possibly safer than CW-TSCPC alone and may more closely approximate the safety profile of MP-TSCPC.

Additionally, our Kaplan–Meier analysis demonstrated a high cumulative success rate of 80.8% at POY1 given our success criteria of IOP reduction ≥ 30%, which was more strict than the 20% IOP reduction criteria used by many other papers [26]. When an even stricter criteria of 50% IOP reduction was applied to our data, the cumulative success rate was 71.6% at POY1, higher than the 59.6% success rate at POY1 achieved by Garcia et al. using only MP-TSCPC and a less restrictive IOP reduction criteria of 20% [26]. It has been well-documented that the IOP-lowering effect of MP-TSCPC appears to wane over time [1]. For example, while Zarrour et al. reported that 86.7% of patients undergoing MP-TSCPC achieved surgical success at 1 month post-treatment (defined as IOP reduction ≥ 20% from baseline), success rates decreased progressively to 67.1% at 6 months and 56.7% in 1 year [27]. A similar decline in success over time was noted in our results, decreasing from 80.8 at POY1 to 65% POY1.5 using a more stringent success criteria of IOP reduction ≥ 30%. Interestingly, it appears that patients treated with combined MP-CPC and CW-TSCPC did not experience a decline in success that was as drastic as those seen in other studies. These results again suggest a significant and more sustained benefit in IOP reduction from our combined procedure involving MP-TSCPC and CW-TSCPC compared to MP-TSCPC alone.

Considering that baseline IOP has been shown to correlate with the magnitude of IOP reduction, it is also important to note our significant IOP reduction of 15.1 mmHg at POY1 in relation to our average preoperative IOP of 27.9 mmHg [24]. In comparison, Vernon et al. demonstrated an average IOP reduction of 10.67 mmHg in their cohort with preoperative IOP ≤ 30 mmHg [6]. This correlation between baseline IOP and magnitude of IOP reduction was present in our Cox proportional hazard regression analysis, where every one-unit increase in preoperative IOP reduced the hazard of clinical failure by 14–15% (depending on whether the IOP reduction ≥ 30% or IOP reduction ≥ 50% success criteria was utilized). This suggests that a higher preoperative IOP is associated with a larger IOP reduction postoperatively and higher likelihood of clinical success.

Altogether, the safety and efficacy findings from the combined procedure suggest that blending the safety profile of MP-TSCPC with the IOP-lowering efficacy of CW-TSCPC may lead to effective IOP reduction without sight-threatening complications. It makes sense that complementing MP-TSCPC with a second, mechanistically different cyclophotogoaluative modality to target the ciliary body would lead to more definitive inhibition of aqueous humor production than MP-TSCPC alone. Furthermore, a limited application of CW-TSCPC in the combined procedure may likely lead to less dissipation of laser energy to surrounding tissues and therefore fewer side effects than those seen in full application of CW-TSCPC [1]. The methodology of this combined procedure, particularly the specific number of CW-TSCPC spots that should be applied based on preoperative IOP, can likely be further optimized through additional studies to minimize off-target effects and enhance the safety profile even further.

The limitations of this study include small sample size, a retrospective design, complex patient population, lack of a control group for direct comparison, and short follow-up time period for some patients. The wide range in follow-up lengths was likely attributable to the fact that some patients required a second glaucoma procedure soon after the first combined procedure, and these patients are censored from all analyses following the second procedure, thus limiting the follow-up length to that time point. Additionally, we were unable to find a similar control group for comparison for two reasons: (1) the population of patients who have undergone MP-TSCPC with these settings at our institution was limited; and (2) most of the patients in this study have a complex glaucoma history involving multiple mechanisms as well as failed medications and procedures. Together, these limitations may affect generalizability of the results. This risk for selection bias was mitigated in the study design by recruiting all patients who underwent combined treatment with MP-TSCPC and CW-TSCPC over a 17-month window instead of randomly sampling patients during this period.

The patient population in our study had refractory glaucoma, as evidenced by the large proportion of patients with severe (81.6%) and mixed-mechanism glaucoma (50.0%), elevated IOP (up to 47 mmHg), high preoperative medication burden (mean of 3.8 ± 1.2 medications), and a history of failed IOP-lowering procedures. Furthermore, given that our institution is a tertiary referral center, the patients in this study likely had more complex glaucoma than patients in a non-tertiary setting. Nevertheless, our combined procedure achieved significant reductions in IOP and medication burden while preserving long-term VA in our patients.

In summary, our results confirm that this combined treatment with MP-TSCPC and CW-TSCPC is a relatively safe and effective procedure that should be considered in patients with refractory glaucoma. Our findings are promising and point to a significant IOP-lowering effect and decrease in medication burden without greater risk of postoperative complications. Moreover, our combined procedure did not have any sight-threatening complications, given no significant decreases in VA at any follow-up time point. Further studies exploring a larger cohort of patients across multiple providers over a longer period of time would allow us to better characterize the longer-term effects of this combined procedure and determine the optimal number of CW-TSCPC spots for a given IOP. Likewise, a prospective study comparing CW-TSCPC, MP-TSCPC, and the combination of the two procedures would be very helpful to directly compare the outcomes of these procedures.

## Data Availability

Data is available upon request.

## Code availability

Code used for statistical testing is available upon request.

## References

[1] Anand N, Klug E, Nirappel A, Valle DS (2020). A review of cyclodestructive procedures for the treatment of glaucoma. Seminars in Ophthalmology.

[2] Kramp K, Vick HP, Guthoff R (2002). Transscleral diode laser contact cyclophotocoagulation in the treatment of different glaucomas, also as primary surgery. Graefe’s Arch Clin Exp Ophthalmol.

[3] Egbert PR, Fiadoyor S, Budenz DL, Dadzie P, Byrd S (2001). Diode laser transscleral cyclophotocoagulation as a primary surgical treatment for primary open-angle glaucoma. Arch Ophthalmol.

[4] Schlote T, Derse M, Zierhut M (2000). Transscleral diode laser cyclophotocoagulation for the treatment of refractory glaucoma secondary to inflammatory eye diseases. Br J Ophthalmol.

[5] Schlote T, Derse M, Rassmann K, Nicaeus T, Dietz K, Thiel HJ (2001). Efficacy and safety of contact transscleral diode laser cyclophotocoagulation for advanced glaucoma. J Glaucoma.

[6] Vernon SA, Koppens JM, Menon GJ, Negi AK (2006). Diode laser cycloablation in adult glaucoma: long-term results of a standard protocol and review of current literature. Clin Exp Ophthalmol.

[7] Rotchford AP, Jayasawal R, Madhusudhan S, Ho S, King AJ, Vernon SA (2010). Transscleral diode laser cycloablation in patients with good vision. Br J Ophthalmol.

[8] Aquino MCD, Barton K, Tan AMWT, Sng C, Li X, Loon SC, Chew PTK (2015). Micropulse versus continuous wave transscleral diode cyclophotocoagulation in refractory glaucoma: a randomized exploratory study. Clin Exp Ophthalmol.

[9] Abdelrahman AM, El Sayed YM (2018). MicroPulse versus Continuous Wave Transscleral Cyclophotocoagulation in refractory pediatric glaucoma. J Glaucoma.

[10] Kaba Q, Somani S, Tam E, Yuen D (2020). The effectiveness and safety of micropulse cyclophotocoagulation in the treatment of ocular hypertension and glaucoma. Ophthalmol Glaucoma.

[11] Nirappel A, Klug E, Chang EK, Chachanidze M, Anand N, Hall N, Chang TC, Solá-Del Valle D (2020). Augmented MP-TSCPC for the management of elevated IOP in glaucomatous eyes. J Clin Ophthalmol.

[12] Fellman R, Mattox C, Ross K (2011) Know the new glaucoma codes. EyeNet Magazine 65–66. http://www.aao.org/eyenet/article/know-new-glaucoma-staging-codes?october-2011. Accessed 13 Aug 2021

[13] Shaarawy T, Grehn F, Sherwood M, World Glaucoma Association (2009) WGA guidelines on design and reporting of glaucoma surgical trials (Guidelines on Design and Reporting of Glaucoma Surgical Trials). Kugler Publications BV / Amsterdam

[14] Chang EK, Gupta S, Chachanidze M, Miller JB, Chang TC, Solá-Del Valle D (2021) Combined pars plana glaucoma drainage device placement and vitrectomy using a vitrectomy sclerotomy site for tube placement: a case series. BMC Ophthalmol 21(106) 10.1186/s12886-021-01872-z PMC7905981 33632169

[15] Souissi S, Le Mer Y, Metge F, Portmann A, Baudouin C, Labbé A, Hamard P (2020) An update on continuous-wave cyclophotocoagulation (CW-CPC) and micropulse laser treatment (MP-TLT) for adult and pediatric refractory glaucoma. Acta Ophthalmol (Copenh). Online ahead of print. 10.1111/aos.14661 10.1111/aos.14661 33222409

[16] Grueb M, Rohrbach JM, Bartz-Schmidt KU, Schlote T (2006). Transscleral diode laser cyclophotocoagulation as primary and secondary surgical treatment in primary open-angle and pseudoexfoliative glaucoma. Graefe’s Arch Clin Exp Ophthalmol.

[17] Iliev ME, Gerber S (2007). Long-term outcome of trans-scleral diode laser cyclophotocoagulation in refractory glaucoma. Br J Ophthalmol.

[18] Nabili S, Kirkness C (2004). Trans-scleral diode laser cyclophoto-coagulation in the treatment of diabetic neovascular glaucoma. Eye.

[19] Ramli N, Htoon HM, Ho CL, Aung T, Perera S (2012). Risk factors for hypotony after transscleral diode cyclophotocoagulation. J Glaucoma.

[20] Singh K, Jain D, Veerwal V (2017). Diode laser cyclophotocoagulation in Indian eyes: efficacy and safety. Int Ophthalmol.

[21] Chen J, Cohn RA, Lin SC, Cortes AE, Alvarado JA (1997). Endoscopic photocoagulation of the ciliary body for treatment of refractory glaucomas. Am J Ophthalmol.

[22] Yildirim N, Yalvac IS, Sahin A, Ozer A, Bozca T (2009). A comparative study between diode laser cyclophotocoagulation and the Ahmed glaucoma valve implant in neovascular glaucoma: a long-term follow-up. J Glaucoma.

[23] Noureddin BN, Zein W, Haddad C (2006). Diode laser transcleral cyclophotocoagulation for refractory glaucoma: a 1 year follow-up of patients treated using an aggressive protocol. Eye.

[24] Emanuel ME, Grover DS, Fellman RL, Godfrey DG, Smith O, Butler MR, Kornmann HL, Feuer WJ, Goyal S (2017). Micropulse cyclophotocoagulation: initial results in refractory glaucoma. J Glaucoma.

[25] Kuchar S, Moster MR, Reamer CB, Waisbourd M (2016). Treatment outcomes of micropulse transscleral cyclophotocoagulation in advanced glaucoma. Lasers Med Sci.

[26] Garcia GA, Nguyen CV, Yelenskiy A, Akiyama G, McKnight B, Chopra V, Lu K, Huang A, Tan JCH, Francis BA (2019). Micropulse transscleral diode laser cyclophotocoagulation in refractory glaucoma. Ophthalmology Glaucoma.

[27] Zaarour K, Abdelmassih Y, Arej N, Cherfan G, Tomey KF, Khoueir Z (2019). Outcomes of micropulse transscleral cyclophotocoagulation in uncontrolled glaucoma patients. J Glaucoma.

